# *Sortilin 1* Promotes Hepatocellular Carcinoma Cell Proliferation and Migration by Regulating Immune Cell Infiltration

**DOI:** 10.1155/2022/6509028

**Published:** 2022-07-08

**Authors:** Yan Gao, Yan Li, Ziyan Song, Zhenxing Jin, Xiao Li, Chunluan Yuan

**Affiliations:** ^1^Department of Oncology, The First People's Hospital of Lianyungang, Lianyungang 222061, China; ^2^Department of Pharmacy, The First People's Hospital of Lianyungang, Lianyungang 222061, China

## Abstract

**Objectives:**

Recent evidence suggests that *Sort1* promotes carcinogenesis and tumor progression in multiple types of cancers. This study investigates the role of *Sort1* in hepatocellular carcinoma (HCC).

**Methods:**

The differentially expressed gene was screened through GEO and TCGA databases. The *Sort1* gene was identified and its expression was then verified by TCGA and HCCDB (a database of hepatocellular carcinoma expression atlas) databases. The Human Protein Atlas database was used to assess the gene expression in tissues. The TCGA and KM-plotter databases were used to study the relationship between *Sort1* and HCC. The correlation between *Sort1* and immune cells was evaluated through the TIMER database. GO and KEGG enrichment analysis was used to investigate the possible mechanism. The role of *Sort1* in cell proliferation and invasion of HCC was further explored through in vitro experiments.

**Result:**

The differentially expressed molecule obtained from database screening was *Sort1*. Its expression was higher in cancer tissues than in paracancerous ones, and it was mainly located in the cytoplasm. The TCGA, KM-plotter databases, and our study data showed that low expression *of Sort1* in HCC patients had better overall survival (OS), progression-free survival (PFI), and disease-specific survival (DSS). Further analysis indicated a significant correlation between *Sort1* expression and immune cell infiltration. The gene set enrichment analysis (GSEA) analysis showed that *Sort1* affected the biological events of HCC by participating in the WNT, TGF-BETA, JAK, STAT, and CALCIUM signaling pathways. In vitro, cytological experiments demonstrated reduced expression of PCNA, Ki-67, Vimentin, N-cadherin, and MMP-9 mRNA after knocking down *Sort1*, although E-cadherin expression was promoted. Overall, these processes reduced the ability of proliferation and invasion of HCC cells.

**Conclusion:**

Downregulation of *Sort1* can prolong the OS, PFI, and DSS of HCC patients. Furthermore, due to its link with immune cell infiltration, the *Sort1* gene represents a potentially novel predictive biomarker of HCC. The growth of HCC can be significantly inhibited by interfering with *Sort1*; therefore, these results provide a potential target for developing anticancer strategies for HCC.

## 1. Introduction

Hepatocellular carcinoma (HCC) is a type of primary liver cancer that ranks sixth in incidence among all malignant tumors and third in mortality worldwide [1, 2]. The occurrence of hepatocellular carcinoma is a complex process involving multiple genes and steps that are linked to risk factors such as alcohol consumption, aflatoxin, nonalcoholic fatty liver disease, and hepatitis B and C viruses [3, 4]. Diagnosis for HCC usually occurs during the late stages of the condition, resulting in a poor prognosis. At present, HCC is mostly treated by surgery, intrahepatic intervention, targeted therapy, and others. Although these commonly used clinical treatments can prolong the survival time of patients, they have limitations and could not significantly reduce the recurrence and mortality of liver cancer [1, 5]. The occurrence and development of HCC have been found to be linked with a variety of oncogenes, tumor suppressor genes, and signaling pathways, with some examples being the RAS mitogen-activated protein kinase (RAS/RAF/MAPK) and the receptor tyrosine kinase signaling pathways [6]. However, ideal tumor markers that would enable HCC to be diagnosed at an early stage or even to predict prognosis are yet to be available, and the molecular pathogenesis is also being poorly understood. Hence, exploring tumor markers that could assist the early diagnosis and prognosis of HCC would be of great clinical significance.

Sortilin 1 (*Sort1*) is an important lipid metabolism regulatory gene. In 2010, through a genome-wide association study (GWAS), the *Sort1* gene was first proved to be related to the metabolism of low-density lipoprotein, and the gene exists in chromosome lp13.3 in [7]. Located in the trans-Golgi network (TGN), the *Sort1* gene is largely involved in the directional transport of various proteins in lysosomes, although part of *Sort1* can also occur on the plasma membrane where it is involved in receptor-mediated endocytosis [8]. Transformed cells display rewired metabolism, with an increased rate of lipid synthesis being a key feature of this altered metabolism. In this context, aberrant lipid biosynthesis is involved in cancer migration, invasion, and the induction of tumor angiogenesis [9]. Previous studies have demonstrated the link of abnormal lipid metabolism with tumor occurrence and development. For example, Broadfield et al. [10] showed that fat induces glucose metabolism in nontransformed hepatocytes and promotes liver tumorigenesis; Yang et al. [11] showed that miR-760 negatively drives fat metabolism by targeting c-Myc and exerts an anticancer effect in esophageal squamous cell carcinoma. As the liver is an important organ for lipid metabolism in the human body, it is meaningful to study hepatocellular carcinoma from the perspective of lipid metabolism-related genes.

We screened the TCGA database and found that the *Sort1* gene is differentially expressed, which is linked to HCC prognosis. We evaluated the expression of the gene and its role in predicting the survival rate of HCC patients. In addition, the effects of *Sort1* on tumor cell behavior and the underlying mechanisms were uncovered through bioinformatics analyses and in vitro experiments.

## 2. Materials and Methods

### 2.1. Data Analysis

Using the GEOquery package from the GEO database (https://www.ncbi.nlm.nih.gov/geo/query/acc.cgi), two RNA expression datasets, GSE84402 and GSE89377 (containing normal and tumor tissues), were downloaded. The probes corresponding to multiple molecules for one probe were removed. When encountering probes corresponding to the same molecule, only the probe with the largest signal value is retained. The differentially expressed genes dataset of hepatocellular carcinoma was then obtained from the TCGA database (https://tcga-data.nci.nih.gov/) before making a Venn diagram based on the intersection of the three datasets. Eventually, differentially expressed genes related to HCC prognosis were identified after applying a ∣log2FC∣ > 1 and a *p* value < 0.05 as parameters. The result is *Sort1*.

To investigate how *Sort1* and other clinical characteristics, such as age, gender, and disease stage, influenced HCC prognosis, a forest map and both univariate and multivariate Cox regression analyses were applied to display the 95% confidence interval (CI) of each variable, the Hazard Ratio (HR), and the *p* values. The “Survminer” and “Survival” packages in *R* (Version 4.0.3) were implemented for analyzing Kaplan–Meier (KM) survival curves, with the latter subsequently generated with the Kaplan–Meier Plotter (https://kmplot.com). Basically, this involved applying the log-rank test for gene expression in liver cancer to produce *c* curves. The risk score and predictive accuracy of *Sort1* were eventually compared by TimeROC analysis [12–14].

The Tumor Immunity Estimation Resource (TIMER) (https://cistrome.shinyapps.io/Timer) was used to determine how immune cell infiltration in HCC patients was related to the transcription level of *Sort1*. In addition, differentially expressed genes related to *Sort1* gene transcription were analyzed using LinkedOmics (https://www.linkedomics.org/login.php) functional module.

### 2.2. Cell Lines

This study used five hepatocellular carcinoma cell lines (Huh7, HepG2, Hep3B, LO2, and MHCC97H) obtained from the Shanghai Chinese Academy of Sciences Cell Bank.

### 2.3. Reverse Transcription Quantitative PCR Method to Detect *Sort1*-Encoding mRNA

After grinding 100 mg of tumor and paracancerous tissues, obtained from the patients pathologically diagnosed with primary liver cancer included in the study, total RNA was extracted with 1 ml of Trizol lysis solution. From the resulting RNA, a corresponding cDNA was synthesized with the reverse transcription kit before performing real-time fluorescence quantitative PCR. For this purpose, the following primers were used for *Sort1*: forward: 5′-CAGTCCAAGCTATATCGAAG TGAGG-3′; reverse: 5′-AAGATGGTGTTGTCTG ATCCCCATTT-3′; *β-actin*, 5′-AGCCTCGCCT TTGCCGA-3′ and 5′-CTGGTGCCTGGGG CG-3′ were selected as forward and reverse primers, respectively. Finally, the relative expression of the targeted genes was determined based on the 2^−△△Ct^ method to obtain the transcription level of *Sort1* in both sets of tissues.

### 2.4. In Vitro Cytological Experiments

#### 2.4.1. CCK Assay for Cell Viability

The Cell Counting Kit-8 Assay (CCK8) was used as specified by the manufacturer to quantify cell proliferation. After seeding 1500 cells into a 96-well plate, the CCK-8 solution was added to each well on the following day. This was followed by a 4 h incubation under 5% CO_2_ at 37°C before recording light absorbance values at 450 nm with a microplate reader (BioRad). The experiment was repeated three times to obtain the mean values of the three experiments.

#### 2.4.2. Colony Formation Assay

For this assay, after seeding the cells (500 cells/well) into 6-well culture plates, the cells were gently shaken prior to incubation for 10 days at 37°C and 5% CO_2_. After incubation, removal of the medium was followed by cell staining using 0.1% crystal violet (Sigma, St. Louis, MO). Cells were observed under a microscope and the number of positive colonies (>40 *μ*m in diameter) was counted. The experiment was repeated three times, with the colony-forming ability of each cell type recorded each time.

#### 2.4.3. Transwell Migration Assay

Cell migration was analyzed using Transwell chambers (Bd Biosciences, San Jose, CA, USA). To the upper chamber, 200 ul of serum-free DMEM containing 5 × 10^4^ cells was added, while to the lower one, DMEM containing 10% FBS was added. This was followed by a 24 h incubation, after which invading cells on the underside of the membrane were fixed with methanol before staining using crystal violet (Beyotime). An inverted microscope was used to capture Images, with invading cells counted at three different positions. The experiment was performed in triplicate, and the average value of the three experiments was taken.

#### 2.4.4. Transwell Invasion Assay

In this assay, serum-free DMEM at 4°C was used to dilute Matrigel (BD Biosciences, San Jose, USA) 1 : 8 before using the mixture with 50 *μ*l coated polycarbonate filters (8 *μ*m; Corning, NY, USA). After overnight incubation at 37°C, 5 × 10^5^cells, in 200 *μ*l of serum-free DMEM, were seeded into the upper chamber, while to the lower one, 500 *μ*l of DMEM, supplemented with 10% FBS, was added. The cells were allowed to grow under 5% CO_2_ at 37°C, and after 24 h, paraformaldehyde was used to fix the upper chamber prior to staining using 0.5% crystal violet. Eventually, noninvading cells were removed and surface cells were counted under a microscope.

### 2.5. Western Blot Detection

PBS at 4°C was used to wash the cells twice before performing cell lysis in cold RIPA buffer to which protease inhibitors had been added. The extracted proteins had their concentrations determined using the BCA protein assay kit (Pierce, Rockford, IL, USA) before denaturing. Proteins were separated in 10% SDS-PAGE and transferred to a nitrocellulose membrane. Membrane blocking was first carried out at room temperature for 1 h using 5% nonfat milk in Tris-buffered saline containing 0.1% Tween-20 (TBST). After overnight incubation at 4°C with the primary antibody, the membranes were washed three times with TBST before a second 1 h incubation at room temperature with the secondary antibody (anti-rabbit IgG). TBST was finally used to wash the membrane three times before visualizing the target protein using an ECL reagent (EMD Millipore, MA, USA).

### 2.6. Statistical Analysis

For statistical analyses, SPSS (version 24.0; SPSS, Inc., Chicago, IL, USA) and R (version 4.0.1; https://www.r-project.org/) were used. Results for continuous data were first expressed as the mean ± standard deviation, with the significance of differences in means assessed by Student's *t*-tests. Differences in *Sort1* expression between normal and tumor tissues were determined by Wilcoxon's tests, with the Kruskal–Wallis test also used to assess the association of clinical stage and *Sort1* expression. Kaplan–Meier curves were used to determine survival outcomes, and correlations were evaluated based on Spearman's correlation coefficient. For all tests, comparisons were considered to be statistically significant at *p* < 0.05.

## 3. Results

### 3.1. *Sort1* Expression Was Elevated in Hepatocellular Carcinoma

**Figure 1:**
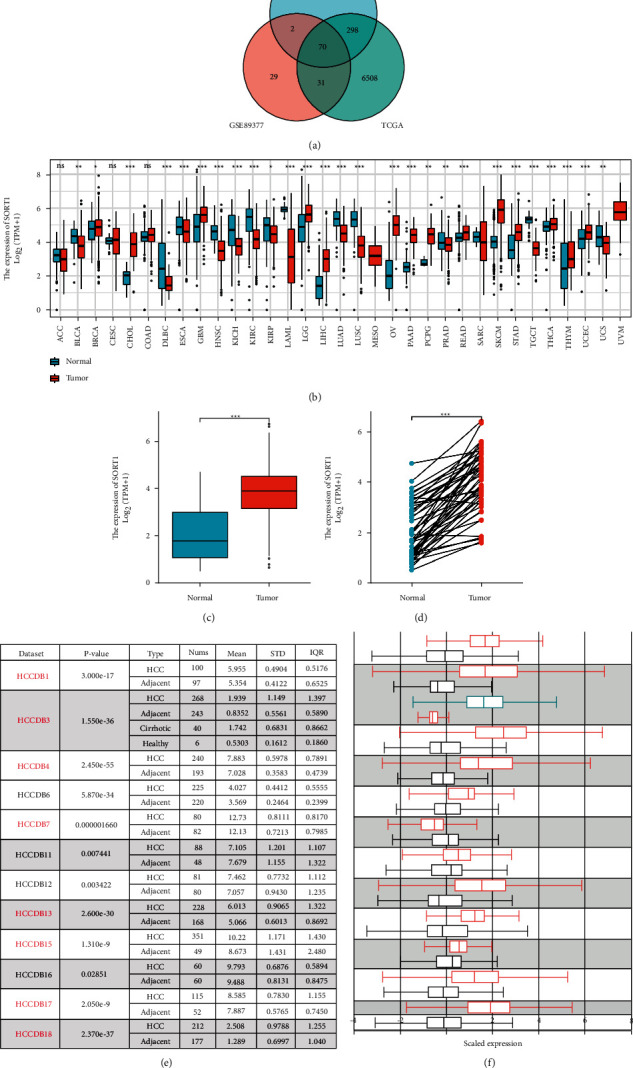
Expression of *Sort1* was elevated in hepatocellular carcinoma. (a) Venn diagrams of genes from the three datasets. (b) *Sort1* expression in pan-cancer analysis. (c) *Sort1* expression in tumor tissues was high for unpaired HCC (*n* = 398) and paracancerous tissues (*n* = 50) in the TCGA database. (d) *Sort1* expression in tumor tissues was high for paired hepatocellular carcinoma (*n* = 50) and paracancerous tissues (*n* = 50) in the TCGA database. (e, f) For HCCDB, significantly higher *Sort1* transcription was observed in HCC tissues as opposed to adjacent normal ones. Differences were statistically significant at ^*∗*^*p* < 0.05, ^∗∗^*p* < 0.01, and ^*∗∗∗*^*p* < 0.001.

We first obtained the differentially expressed gene *Sort1* through Venn diagram analysis between the two datasets, GSE84402 and GSE89377, and the TCGA database (Figure 1(a)). At the same time, it was found by pan-cancer analysis that the *Sort1* gene has high expression and low expression in all tumors (Figure 1(b)). Searches made on the TCGA database indicated that *Sort1* was upregulated in HCC tumor tissues compared with paracancerous ones (Figures 1(c) and 1(d)), with similar differences in mRNA levels found after 12 HCC research cohorts from the HCCDB database were analyzed (Figures 1(e) and 1(f)).

### 3.2. Expression of *Sort1* at Tissue Level in the Human Protein Atlas Database

**Figure 2:**
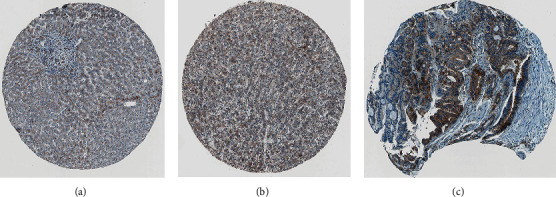
Expression of *Sort1* at the tissue level in the Human Protein Atlas database. Immunohistochemical staining of (a) *Sort1* in normal tissues; (b) low *Sort1* expression in HCC tissues; and (c) *Sort1* expression in HCC tissues.

After analyzing the Human Protein Atlas database to determine *Sort1* expression, it was observed that the gene was mostly located in the cell cytoplasm (Figures 2(a) and 2(c)). The results of immunohistochemistry further indicated that *Sort1* was more expressed in the cancer tissues, especially in poorly differentiated tumors, compared with the paracancerous ones.

### 3.3. Assessing the Prognostic Value of *Sort1* in HCC

**Figure 3:**
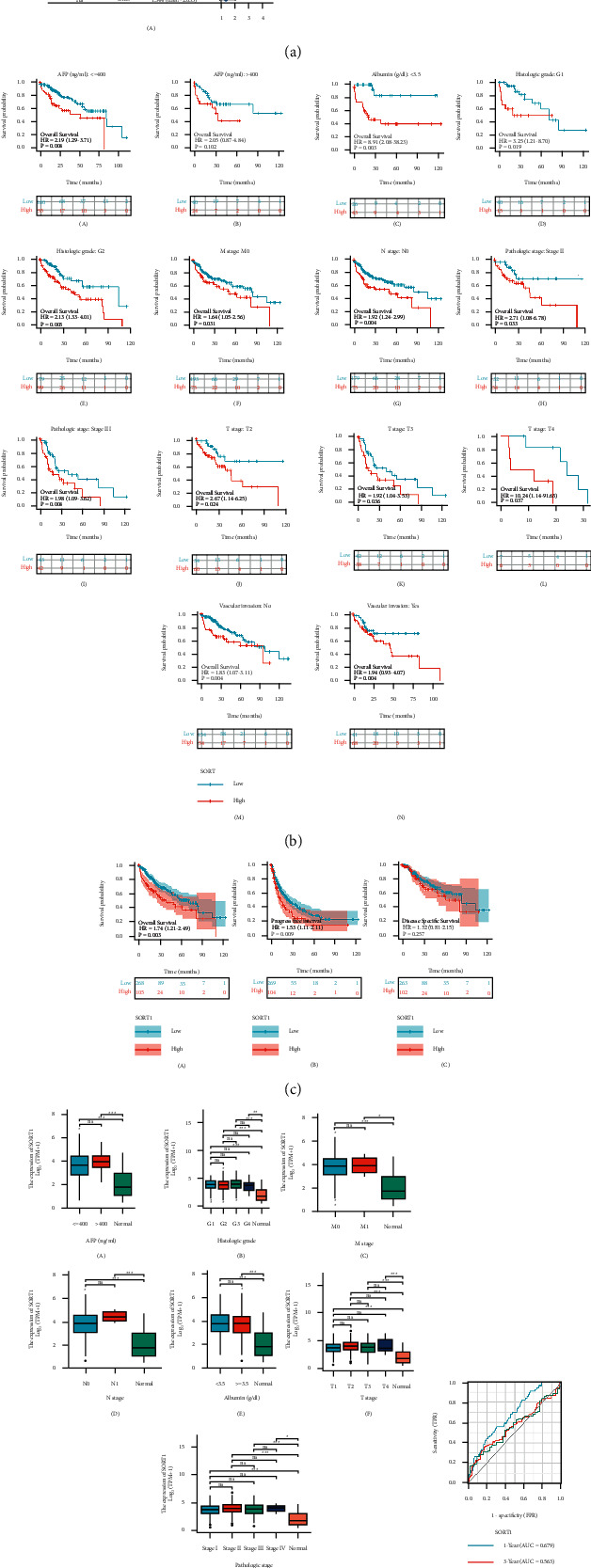
Poor survival was predicted for hepatocellular carcinoma patients with high *Sort1* expression. (a) Forest plot analysis of risk factors for HCC overall survival. (b) The relationship between OS and *Sort1* expression for different groups of HCC patients was stratified using the Kaplan–Meier curve. (c) Overall survival (OS), progression-free survival (PFI), and disease-specific survival (DSS) in relation to *Sort1* expression in HCC patients. (d) *Sort1* expression was correlated with AFP level, tumor stage, and albumin level in hepatocellular carcinoma. (e) Transient ROC analysis of *Sort1* expression in HCC. “ns” means *p* < 0.05; ^*∗*^*p* < 0.05, ^∗∗^*p* < 0.01, ^*∗∗∗*^*p* < 0.001, and ^*∗∗∗∗*^*p* < 0.0001.

To investigate the association of *Sort1* expression and clinical data (age, pathology classification (pTNM, including pT, pN, and pM stages), tumor grades, AFP, albumin level, and presence or absence of vascular invasion) and OS in HCC patients, univariate and multivariate Cox regression analyses were used. A significant association between pM stage (*p* value = 0.017), pT stage (*p*.value < 0.001), *Sort1* expression (*p* value = 0.048), and OS was found based on univariate Cox analysis. Multivariate analysis also highlighted the significance of *Sort1* expression (*p* value = 0.008), indicating that *Sort1* could be a prognostic factor for HCC (Figure 3(a)). On stratifying clinical factors using Kaplan–Meier (KM) plots, it was observed that low *Sort1* expression was a better prognostic factor. These results were supported by previous reports that *Sort1* is an “oncogene” in HCC (Figure 3(b)). *Sort1* expression was also related to survival outcomes of the HCC cohort based on the Kaplan–Meier plotter's liver cancer RNA-seq database and plotted Kaplan–Meier survival curves as in this case. High *Sort1* expression had poorer OS, PFI, and DSS than those with low expression. In particular, OS and PFI had statistical significance (*p* < 0.05) (Figure 3(c)). In addition, as AFP, pT, pN, and pM stages of HCC increased, the *Sort1* expression decreased (Figure 3(d)), suggesting that *Sort1* could potentially act as a biomarker for HCC disease progression. Furthermore, risk score and the predictive accuracy of ASF1B were compared by ROC analysis. The results showed that *Sort1* expression can predict the 1-year, 3-year, and 5-year survival. The AUC under the ROC curve was 0.679, 0.563, and 0.558, respectively (Figure 3(e)). These findings indicate that *Sort1* has a predictive role for the prognosis of HCC.

### 3.4. *Sort1* Expression and Immune Cells Infiltration Based on the TIMER Database

**Figure 4:**
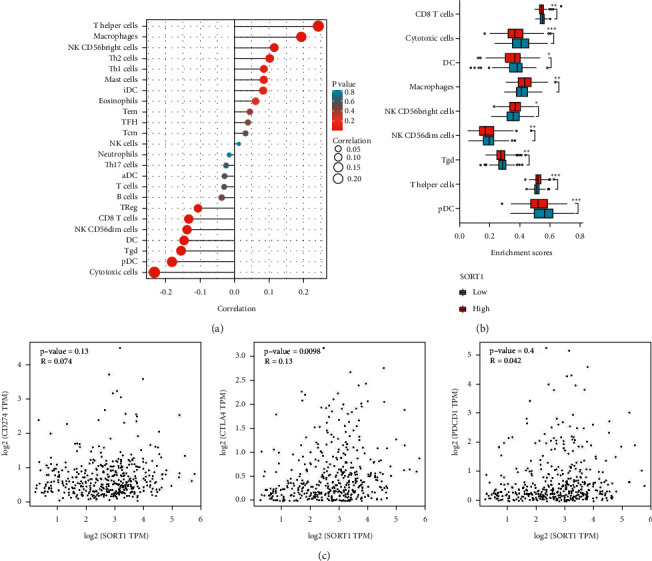
*Sort1* expression and immune cell infiltration based on the TIMER database. (a) Positive correlation between immune cells infiltration and *Sort1* in the TIMER database. (b) Significant correlation between infiltration of immune cells in HCC and *Sort1* expression. (c) Scatter plot showing *Sort1* correlation with CTLA-4, CD274, and PDCD1.

In order to assess the association of Sort1 expression and different types of immune cell infiltration in HCC, bar graphs from the TIMER database were constructed. Overall, the gene's expression was found to be positively correlated with some of the most infiltrating immune cells, such as mast cells, Th1 cells, Th2 cells, NK CD56 bright cells, macrophages, and *T* helper cells (Figure 4(a)). The influence of *Sort1* on the tumor microenvironment (TME) was further assessed by determining the relationship between specific immune cells and *Sort1*. The results showed a positive correlation between the gene and the infiltration levels of *T* helper cells, NK CD56 bright cells, and macrophages. However, a negative correlation was observed with the infiltration levels of DCs, cytotoxic T cells, NK CD56dim cells, Tgd, and pDCs (Figure 4(b)). Furthermore, the results also indicated good correlations between *Sort1* expression and molecules such as PDCD1, CD274, and CTLA-4 that are involved in immune checkpoints (Figure 4(c)). Altogether, these results suggest a certain correlation between *Sort1* expression and immune cell infiltration, with the tumor microenvironment of HCC likely to be involved in allowing cancer cells to evade the immune system. These findings can form the basis of future research.

### 3.5. The Coexpression Network of *Sort1* Suggests a Potential Function of *Sort1* in HCC

**Figure 5:**
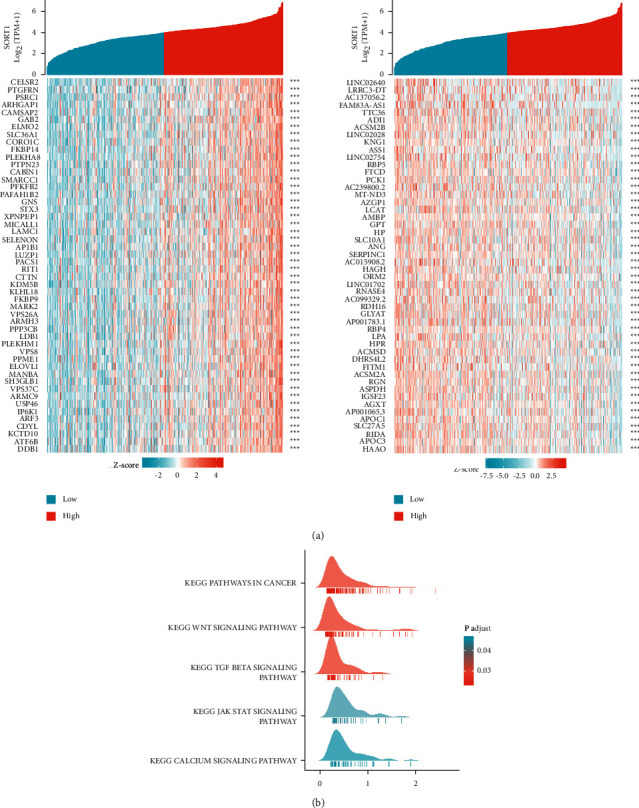
Analysis of genes coexpressed with *Sort1* (LinkedOmics). (a) Cotranscribed genes that were upregulated or downregulated by *Sort1*. (b) Ridge plot of GSEA analysis.

The coexpression network of *Sort1* in the hepatocellular carcinoma (HCC) group was analyzed using LinkedOmics to determine the biological significance of the gene. Genes that were positively or negatively correlated with *Sort1* expression were shown in the heatmap (Figure 5(a)). By analyzing these genes, it was found that *Sort1* is associated with the upregulation of HCC risk factors while downregulating those that protect against HCC. In addition, *Sort1* is involved in HCC occurrence and development. Gene set enrichment analysis (GSEA) further showed that *Sort1* could affect HCC prognosis by influencing the WNT, TGF-BETA, JAK, STAT, and CALCIUM signaling pathways (Figure 5(b)).

### 3.6. Effects of *Sort1* Knockdown on Proliferation and Clonogenic Ability of HCC Cells

**Figure 6:**
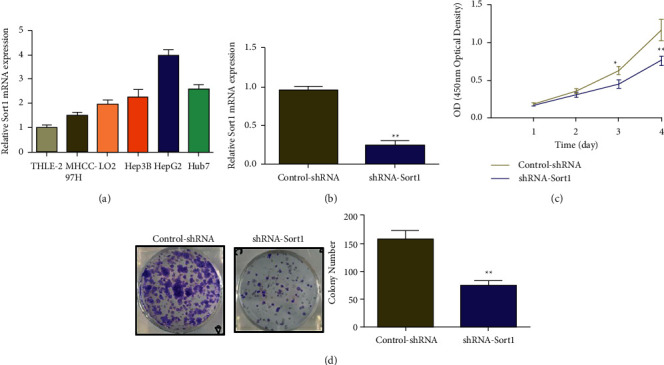
Knocking down *Sort1* significantly inhibited proliferation and colony formation in HCC cells. (a) *Sort1* expression levels in HCC cell lines and human normal hepatocytes (THLE-2) determined by real-time PCR. (b) Effects of shRNA-*Sort1* transfection on *Sort1* expression in HepG2 cells determined by fluorescent quantitative PCR. (c) Effect of *Sort1* knockdown on HepG2 proliferation determined by CCK-8 assay. (d) Effects of *Sort1* knockdown on the clonogenic ability of HepG2 cells based on the colony formation assay. ^*∗*^*p* < 0.05 and ^∗∗^*p* < 0.01.

Fluorescence quantitative PCR was used to assess *Sort1* expression levels in HCC cell lines and human normal hepatocytes (THLE-2) cells. The expression levels were significantly increased in HCC cell lines (MHCC97H, LO2, Hep3B, HepG2, and Huh7), with the highest expression level being in HepG2 cells (Figure 6(a)). Therefore, in the subsequent knockdown experiments, HepG2 cells were selected. HepG2 was transfected with a lentiviral interference vector (shRNA-*Sort1*) targeting Sort1. The results of quantitative fluorescence PCR showed that the transfection of shRNA-*Sort1* could significantly reduce *Sort1* expression compared with the control group (Control-shRNA) (Figure 6(b)). Next, the effects of *Sort1* knockdown on the proliferation of hepatoma cells HepG2 were investigated using the CCK-8 assay. In this case, the results indicated the inhibition of HepG2 proliferation after transfection of shRNA-*Sort1* (3 days) compared with the control (Control-shRNA). After 4 days of culture, knockdown of *Sort1* inhibited the proliferation ability of HepG2 cells more significantly (Figure 6(c)). Finally, based on the colony formation experiment, it was observed that the transfection of shRNA-*Sort1* resulted in a significant inhibition in the ability of HepG2 cells to form colonies, especially in comparison to the control (Control-shRNA) (Figure 6(d)).

### 3.7. Effects of *Sort1* Knockdown on the Invasion and Migration of HCC Cells

**Figure 7:**
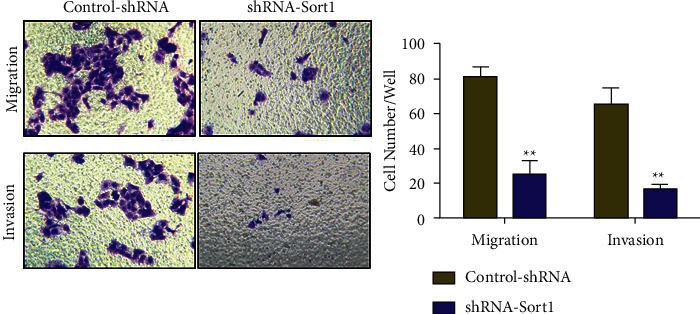
HepG2's invasion and migration abilities were significantly inhibited after *Sort1* knockdown. ^∗∗^*p* < 0.01.

The high mortality of liver cancer can be attributed to metastasis, especially in the advanced stage of liver cancer. Therefore, Transwell was used to determine how *Sort1* influenced the ability of cell invasion and migration. It was found that compared with the control (Control-shRNA), a significant reduction in the migration and invasion abilities of HepG2 cells occurred after transfection with shRNA-*Sort1* (Figure 7).

### 3.8. Effects of *Sort1* Knockdown on the Expression of Molecules Related to Cell Proliferation and Invasion

**Figure 8:**
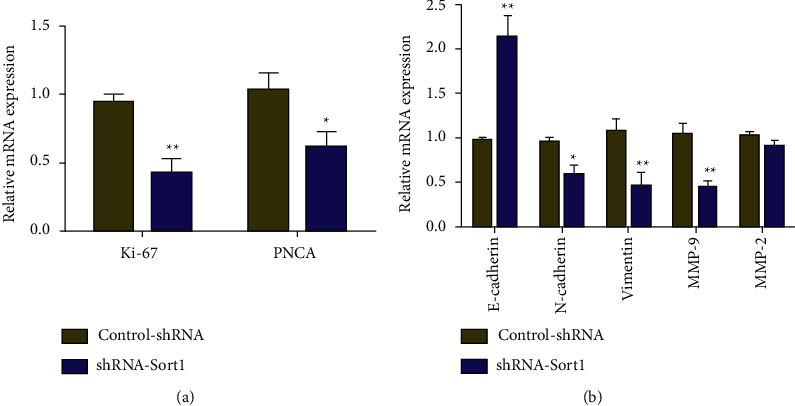
The expression of genes related to proliferation and invasion of HepG2 cells was significantly inhibited by *Sort1* knockdown. ^*∗*^*p* < 0.05 and ^∗∗^*p* < 0.01.

Following the above results, real-time quantitative PCR was applied to examine further the underlying mechanism through which *Sort1* knockdown inhibited the cell proliferation and invasion. The results indicated that, in comparison with the control (Control-shRNA), knocking down *Sort1* inhibited the proliferation and invasion of HCC cells and the resulting low *Sort1* also significantly reduced the mRNA levels of intracellular Ki-67 and PCNA (Proliferating Cell Nuclear Antigen) (Figure 8(a)). In addition, as opposed to the control (Control-shRNA), *Sort1* knockdown significantly promoted E-cadherin expression while inhibiting those of MMP-9 mRNA, Vimentin, and N-cadherin. However, the gene knockdown did not affect MMP-2 expression (Figure 8(b)). Thus, the results suggested that the expression of molecules related to invasion and proliferation were significantly inhibited by *Sort1* knockdown.

## 4. Discussion

HCC, as a common primary liver cancer, has increased in prevalence in recent years [15]. Currently, hepatectomy, liver transplantation, and local ablation remain the most effective curative methods, but HCC patients still have a low 5-year survival rate [16, 17]. In fact, by the time they are diagnosed, many HCC patients already reach the middle and advanced stages and often have severe liver cirrhosis, thus making them unsuitable for surgical resection or liver transplantation [18]. In contrast, the more popular immunotherapy can reverse the immune escape of tumors by inhibiting or activating certain immune checkpoints [19, 20]. Relevant clinical studies have confirmed that immune checkpoint therapy is an effective means of treating tumors [21, 22]. Therefore, finding new sensitive molecular markers and therapeutic targets is crucial for improving the prognosis of HCC patients.

As sequencing and omics technologies developed, it became possible to better understand the mechanism of HCC and identify target genes that are of potential diagnostic and therapeutic value [23]. In our study, we screened HCC genes from GEO and TCGA data and identified the differentially expressed molecule *Sort1* from the differential genes. Previous studies have shown that *Sort1* acts as an oncogene that is linked with poor prognosis in gastric [24], prostate [25], and colorectal cancers [26], but there is no relevant study on a similar mechanism in HCC. We verified the ability of reduced *Sort1* expression in inhibiting HCC proliferation and invasion, with the findings expected to reflect the potential importance of using *Sort1* to assess the prognosis of HCC.

We obtained the common upregulated differentially expressed gene *Sort1* by taking the intersection of the three datasets obtained from the GEO and TCGA data and presented them in Venn diagrams. By analyzing *Sort1* expression based on multiple databases and bioinformatics analyses, it was observed that most cancers, including liver cancer, abnormally expressed this gene. This result was supported by existing literature [27–29]. RNA-seq data in TCGA and corresponding clinical data were analyzed to further determine how *Sort1* and HCC were related. In this case, Cox regression analysis demonstrated that *Sort1* could represent a risk factor for HCC prognosis, with high expression of the gene being linked to poor prognosis. The *Sort1* expression was associated with the progression of tumor *T* stage and overall disease progression.

Currently, there is an increasing interest in immunotherapy in the treatment of HCC. Previous studies have shown that tumor-infiltrating lymphocytes independently predict the status of sentinel lymph nodes and the survival of cancer patients [30, 31]. By mining public data, it was found that *Sort1* expression was correlated with immune cell infiltration, with a positive correlation with most infiltrating immune cells such as mast cells, Th1 cells, Th2 cells, NK CD56 bright cells, macrophages, and *T* helper cells among others. In contrast, a negative correlation with the infiltration levels of DC, cytotoxic T cells, NK CD56dim cells, Tgd, and pDC were observed. Furthermore, molecules such as CTLA-4, CD274, and PDCD1 that are involved in immune checkpoints were also correlated with *Sort1* expression. Thus, by highlighting the significant relationship between immune cell infiltration and *Sort1* expression, the results suggest not only that *Sort1* is involved in the HCC tumor microenvironment but also that this process could be important for allowing tumor cells to evade the immune system.

When analyzing genes that were significantly associated with *Sort1* expression in HCC, it was observed that these genes were also abnormally expressed, with most of them being linked with the overall survival of HCC cells. It is quite likely that *Sort1* interacts with these genes to establish a regulatory network that eventually promotes HCC occurrence and development. Gene set enrichment analysis (GSEA) further revealed that *Sort1* could be involved in the WNT, TGF-BETA, JAK, STAT, and CALCIUM signaling pathways, resulting in the different prognosis of HCC, which are also associated with the high proliferation of HCC. The pathological features are consistent with other hyperproliferative cancers [32].

Predicting outcomes and discovering key factors in the biological mechanisms leading to adverse outcomes are two important parts of cancer research [33]. Based on the above research results, we found that *Sort1* may be involved in the poor prognosis of HCC through a certain pathway mechanism. Therefore, we further verified the expression of *Sort1* in HCC through cytological experiments. It was found that *Sort1* expression was higher in liver cancer cell lines (Huh7, HepG2, Hep3B, LO2, and MHCC97H) than that on normal human hepatocytes (THLE-2), with the highest expression level occurring in HepG2 cells. Hence, *Sort1* was knocked down in HepG2 cells, which reduced the proliferation and invasion of the cells, suggesting that this gene is important to maintain tumorigenic activity in vitro. The expression of Ki-67, PCNA, N-cadherin, E-cadherin, Vimentin, and MMP-9 mRNA was assessed by real-time PCR to investigate the underlying mechanism behind HCC suppression after *Sort1* knockdown. Ki-67 is a proliferating cell-associated nuclear antigen. Previous studies have shown that Ki-67 expression and tumor lymph node metastasis are two independent prognostic factors for disease-free survival and overall survival in HCC patients, which may help decision-making of adjuvant therapy [34]. PCNA is an important factor representing DNA replication [35]. Gramantieri et al. [36] suggested that in human hepatocellular carcinoma with cirrhosis, cell proliferation involving P21 during DNA repair depends on PCNA. Gan et al. [37] found that RAR*γ*-induced downregulation of E-cadherin induced HCC cells to invade and metastasize, and tumor metastasis and poor surgical outcome were linked with reduced expression of N-cadherin in cancer cells [38]. Similarly, in HCC, Huang et al. [39] found that CMTM6 interacted with and stabilized vimentin to promote migration, invasion, and Epithelial-Mesenchymal Transition (EMT). Finally, as complex matrix metalloproteinases, MMP-9 is involved in tumor cell invasion and metastasis by degrading extracellular matrix (ECM) components [40]. In this study, knocking down of *Sort1* significantly promoted E-cadherin expression and suppressed the mRNA levels of Ki-67, PCNA, N-cadherin, Vimentin, and MMP-9 mRNA. Based on the results, it is likely that *Sort1* is involved in various pathological events of HCC through its ability to regulate cell proliferation and invasion.

## 5. Conclusion

In conclusion, this study provides various types of evidence to support the significance of *Sort1* in HCC development, especially in its value as a potential biomarker of HCC progression. Interfering with *Sort1* significantly inhibited HCC growth by influencing the ability of cells to proliferate and invade. This study provides a potential target for developing anticancer strategies against HCC.

## Data Availability

The data are available from the corresponding author upon request via e-mail (yuancla@163.com).
